# Trends in a Life Threatening Condition: Morbid Obesity in Dutch, Turkish and Moroccan Children in The Netherlands

**DOI:** 10.1371/journal.pone.0094299

**Published:** 2014-04-14

**Authors:** Paula van Dommelen, Yvonne Schönbeck, Stef van Buuren, Remy A. HiraSing

**Affiliations:** 1 Department of Life Style, TNO, Leiden, The Netherlands; 2 Department of Child Health, TNO, Leiden, The Netherlands; 3 Department of Methodology and Statistics, University Utrecht, Utrecht, The Netherlands; 4 EMGO Institute of Health Care Research, VU University Medical Centre Amsterdam, Amsterdam, The Netherlands; University of Missouri-Kansas City, United States of America

## Abstract

**Background:**

Morbid obesity can be a life threatening condition. The aim of our study is to assess the trend in morbid obesity in The Netherlands among children of Dutch origin since 1980, and among children of Turkish and Moroccan origin since 1997.

**Methods and Findings:**

Cross-sectional height and weight data of children of Dutch, Turkish and Moroccan origin aged 2–18 years were selected from three national Dutch Growth Studies performed in 1980, 1997 and 2009 (n = 54,814). Extended international (IOTF) cut-offs in childhood were used to define morbid obesity (obesity class II and III combined). The morbidity index for overweight was calculated as the prevalence of morbid obesity divided by the prevalence of overweight. Our study showed that the prevalence of morbid obesity in children of Dutch origin was 0.59% in boys and 0.53% in girls in 2009. Significant upward trends occurred since 1980 and 1997. The prevalence was three to four fold higher in Turkish children compared to Dutch children. The Turkish children also had an upward trend since 1997, but this was only statistically significant in boys. The prevalence of morbid obesity in Moroccan children was two to three fold higher than in Dutch children, but it remained almost stable between 1997 and 2009. The Dutch and Turkish children showed an upward trend in morbidity index for overweight since respectively 1980 and 1997, while the Moroccan children showed a downward trend since 1997. In 2009, children of low educated parents had the highest prevalence rates of morbid obesity; 1.06% in Dutch, 2.11% in Turkish and 1.41% in Moroccan children.

**Conclusions and Significance:**

An upward trend of morbid obesity in Dutch and Turkish children in The Netherlands occurred. Monitoring and reducing the prevalence of childhood morbid obesity is of high importance for these children, health care and the community.

## Introduction

Over the past three decades childhood overweight and obesity have reached epidemic proportions in most industrialized countries [1]. Although the Dutch prevalence rates of childhood overweight and obesity are still low compared to other developed countries [2], [3], both rates were higher than in 1980. In 2009, fourteen percent of Dutch children were overweight and two percent were obese [3]. This is a two to three fold higher prevalence in overweight and four to six fold increase in obesity since 1980 [4]. Upward trends were also found in the two largest ethnic minorities in The Netherlands originating from Turkey and Morocco.

Obesity is a leading preventable cause of chronic illnesses and death and has emerged as one of the most serious public health concerns in the 21st century [5]. Excessive body weight is associated with various diseases like cardiovascular diseases, diabetes mellitus type 2, and certain types of cancer [6]. The risks increase with higher BMI levels at the upper end of the distribution [7]. Obesity has been found to reduce life expectancy by two to four years, while morbid obesity even reduces life expectancy by ten years [7]. Therefore, in adults obesity has been broken down into three classes; class I obesity (30.0–34.9 kg/m^2^), class II obesity (35.0–39.9 kg/m^2^) and class III obesity (≥40.0 kg/m^2^). Only recently, in 2012, extended international (IOTF) cut-offs for childhood obesity classes II and III were presented [8]. Also in children, morbid obesity can be a life threatening condition [9]. Childhood morbid obesity often causes hypertension, dyslipidemia, insulin resistance/diabetes, fatty liver disease, and psychosocial complications [10]. In a study in 2005–2007 in The Netherlands among 307 children with severely obesity, 56% had hypertension and 67% had at least one cardiovascular risk factor [11]. A study among 230,000 Norwegian adolescents showed that morbid obesity was associated with increased mortality in middle age from a wide variety of systemic diseases [12].

The aim of our study is to apply the extended international (IOTF) cut-offs on the national Dutch growth studies to assess the trend in morbid obesity (class II and III combined) in The Netherlands among children of Dutch origin since 1980, and among children of Turkish and Moroccan origin since 1997. The Dutch growth studies offer a unique opportunity to study trends covering the time period before which the worldwide obesity epidemic emerged.

## Methods

### Ethics Statement

Data collection of growth studies is part of routine youth health care in The Netherlands, and is not regarded as medical research. In the Dutch nationwide surveys, written informed consent was not needed. Verbal consent was obtained from each child (and parent for children younger than 16 years) before measurement. To document the process, cooperation, or lack thereof, was registered on the questionnaire. The Medical Ethical Review Board of Leiden University Medical Centre approved of the study and the way consent was obtained.

### Data

Cross-sectional height and weight data of children of Dutch, Turkish and Moroccan origin aged 2–18 years living in The Netherlands were selected from the Third Growth Study in 1980 (n = 30,020) [4], the Fourth Growth Study in 1997 (n = 13,900) [13] and the Fifth Growth Study in 2009 (n = 10,894) [3]. Data were obtained at Well Baby Clinics, Municipal Health Services (MHS), schools and a festival (in 1997 and 2009). To obtain sufficient samples of Turkish and Moroccan children, oversampling was done in the four major cities Amsterdam, Rotterdam, The Hague, and Utrecht, where most children of Turkish and Moroccan origin in The Netherlands live. Measurements in all Dutch Growth studies were identical and were standardized and performed by trained health care professionals. Height was measured to the nearest 0.1 cm. Children were weighed, wearing underwear only, or a correction was made for clothes, on calibrated mechanical or electronic step scales. Weight was rounded to the nearest 0.1 kg. Children with diagnosed growth disorders and those on medication known to interfere with growth were excluded from these surveys. More details on the design of these surveys have been published before [3], [4], [13].

### Statistical analyses

Dutch origin was defined as both mother and father born in The Netherlands, Turkish origin was defined as mother born in Turkey, or mother born in The Netherlands and father born in Turkey. Similarly, Moroccan origin was defined as mother born in Morocco, or mother born in The Netherlands and father born in Morocco. Parental education was defined as the educational level of the highest educated parent and categorized into low, medium and high [14]. Prevalence rates for morbid obesity were calculated with the extended international (IOTF) cut-offs (class II and III combined) for children [8]. These cut-offs were obtained by a centile at the growth curve of six large nationally representative cross-sectional growth studies, including The Third Dutch Growth Study in 1980 [4]. At age 18 years, this centile passed through the cut-off point of 35 kg/m^2^ for adults class II obesity. Since the number of children was not evenly distributed across age years, we first calculated the prevalence rates per year of age, and then averaged these rates. Chi-square and Fisher's exact tests were performed to test for differences in prevalence rates. A log-binomial model was used to test if differences in prevalence rates between ethnic groups in 2009 are due to differences in the educational level of the parents, after adjustment for sex and age of the child. P-values <0.05 (two-sided) were considered statistically significant. As the risk of morbidity in overweight children increases with higher BMI, we calculated the morbidity index for overweight. The morbidity index is used in other studies to categorize the severity of a disease, for example diabetes mellitus [15] and coronary risk factors [16]. In our study, the morbidity index for overweight was calculated as the prevalence of morbid obesity divided by the prevalence of overweight expressed as a percentage. The statistical analyses were performed in SPSS version 20.0 for Windows (SPSS Inc., Chicago, IL, USA).

## Results

### Morbid obesity in children of Dutch origin

**Figure 1:**
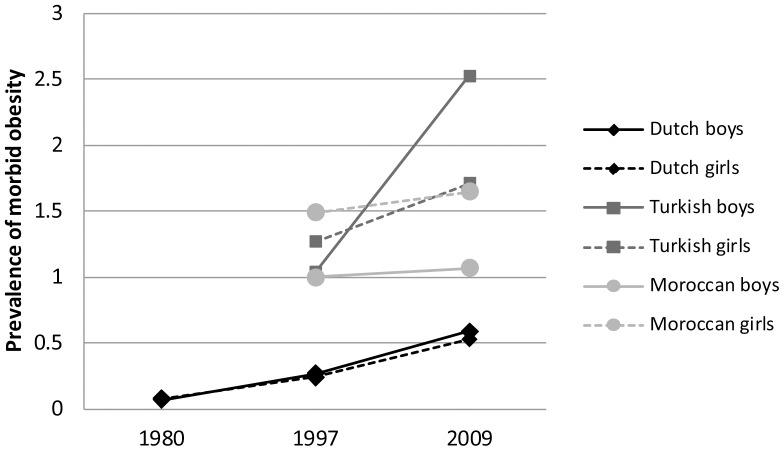
The trend in morbid obesity in The Netherlands among children of Dutch origin since 1980, and among children of Turkish and Moroccan origin since 1997.

Figure 1 and Table 1 show the trend in morbid obesity in The Netherlands among children of Dutch origin since 1980, and among children of Turkish and Moroccan origin since 1997. Table 1 shows that the overall trends and prevalence rates were almost similar between boys and girls of Dutch origin. Between 1980 and 1997 there has been an upward trend in the prevalence of morbid obesity in boys and girls of Dutch origin (respectively, p<0.001 and p<0.01). Upward trends were also found between 1997 and 2009 in boys and girls (both p<0.05). Almost in all age groups, there has been an increase in the prevalence of morbid obesity. In 2009, 0.59% in boys and 0.53% in girls were morbidly obese.

**Table 1:** The trend in morbid obesity in The Netherlands among children of Dutch origin since 1980, and among children of Turkish and Moroccan origin since 1997.

Group	Age (y)	% of morbid obesity (N) in 1980	% of morbid obesity (N) in 1997	% of morbid obesity (N) in 2009
Dutch boys	2–5 y	0.14 (4943)	0.44 (961)	0.53 (757)
	6–11 y	0.12 (4611)	0.37 (1367)	0.62 (1158)
	12–18 y	0.00 (5818)	0.08 (2667)	0.60 (1210)**
	Total	0.07 (15372)	0.27 (4995)***	0.59 (3125)*
Dutch girls	2–5 y	0.32 (4747)	0.37 (980)	1.00 (817)
	6–11 y	0.03 (4609)	0.05 (1361)	0.63 (1318)*
	12–18 y	0.00 (5292)	0.34 (2292)***	0.17 (1360)
	Total	0.08 (14648)	0.24 (4633)**	0.53 (3495)*
Turkish boys	2–5 y		0.37 (194)	4.58 (543)**
	6–11 y		0.84 (400)	2.53 (281)
	12–18 y		1.59 (525)	1.36 (255)
	Total		1.04 (1119)	2.53 (1079)*
Turkish girls	2–5 y		1.06 (156)	1.54 (501)
	6–11 y		0.66 (399)	3.50 (291)*
	12–18 y		1.90 (469)	0.27 (244)
	Total		1.27 (1024)	1.71 (1036)
Moroccan boys	2–5 y		2.66 (179)	1.78 (485)
	6–11 y		0.67 (390)	0.43 (371)
	12–18 y		0.35 (507)	1.22 (204)
	Total		1.00 (1076)	1.07 (1060)
Moroccan girls	2–5 y		2.84 (187)	2.24 (444)
	6–11 y		1.99 (384)	1.25 (403)
	12–18 y		0.29 (482)	1.65 (252)*
	Total		1.49 (1053)	1.65 (1099)

### Morbid obesity in children of Turkish origin

Table 1 shows that the prevalence of morbid obesity is higher in Turkish children compared to the Dutch. An upward trend was noticed in Turkish boys between 1997 and 2009 (p<0.05). In 2009, 2.53% of the Turkish boys were morbidly obese. In 2009, the prevalence in Turkish girls was slightly higher (1.71%) than in 1997 (1.27%). There was a rise in the prevalence up to twelve years of age, significantly in 2–5 year old boys (p<0.01) and 6–11 year old girls (p<0.05).

### Morbid obesity in children of Moroccan origin

The overall prevalence of morbid obesity in Moroccan boys and girls remained almost stable over time (see Table 1). However, in the age group 12–18 years, there was an upward trend in girls (p<0.05) and a (non-significant) upward trend in boys, while the other age groups showed a (non-significant) downward trend. In all age groups, girls had higher prevalence rates than boys in 2009. The overall prevalence of morbid obesity in 2009 was 1.07% in boys and 1.65% in girls.

### Morbidity index for overweight

Table 2 presents the morbidity index for overweight among Dutch children between 1980 and 2009 and among Turkish and Moroccan children between 1997 and 2009. In 2009, the highest morbidity index for overweight was found in Turkish boys (7.76%). Moroccan and Turkish girls had the second and third highest morbidity index for overweight of respectively 5.83% and 5.48%. The lowest morbidity index for overweight (3.61%) was found in Dutch girls. The Dutch and Turkish children showed an upward trend in morbidity index for overweight since respectively 1980 and 1997, while the Moroccan children showed a downward trend since 1997.

**Table 2:** The trend in morbidity index for overweight defined as the prevalence of morbid obesity divided by the prevalence of overweight (%) in The Netherlands among children (2–18 years) of Dutch origin since 1980, and among children of Turkish and Moroccan origin since 1997.

	Boys			Girls		
Ethnicity	MI-O (%) in 1980	MI-O (%) in 1997	MI-O (%) in 2009	MI-O (%) in 1980	MI-O (%) in 1997	MI-O (%) in 2009
Dutch	1.52	3.03	4.72	1.13	2.09	3.61
Turkish		4.37	7.76		4.31	5.48
Moroccan		6.33	4.31		6.39	5.83

MI-O: Morbidity Index for Overweight.

### Morbid obesity and educational level in 2009

In 2009, 18% of Dutch children had low educated parents, while this was 58% in Turkish and 60% in Moroccan children. Dutch children of low educated parents had the highest prevalence of morbid obesity (1.06%), while those of medium and high educated parents had the lowest prevalence rates of morbid obesity (respectively 0.51% and 0.16%). Similar results were found in children of Turkish and Moroccan origin. For Turkish children, the prevalence of morbid obesity was 2.11% in low educated parents, 1.09% in medium, and 1.10% in high educated parents. In Moroccan children, these rates were respectively 1.41%, 0.68%, and 1.16%. After adjustment for educational level, the differences in morbid obesity rates between Turkish and Dutch children (from relative risk (RR) = 4.1, 95%CI(2.7–6.3) to RR = 2.0, 95%CI(0.97–4.3)) and between Moroccan and Dutch children (from RR = 2.7, 95%CI(1.7–4.4) to RR = 1.3, 95%CI(0.5–3.1)) were less pronounced.

## Discussion

This study is the first that presents trends of morbid obesity based on the extended international (IOTF) cut-offs in Dutch children, and also in Turkish and Moroccan children living in The Netherlands. We showed that the prevalence of morbid obesity in children of Dutch origin was 0.59% in boys and 0.53% in girls in 2009. Significant upward trends occurred since 1980 and 1997. The prevalence rates were much higher in children of Turkish (2.53% in boys and 1.71% in girls) and Moroccan (1.07% in boys and 1.65% in girls) origin in 2009. Turkish children showed an upward trend before twelve years of age, while the results of the Moroccan children showed the opposite; an upward trend aged twelve years onwards. The morbidity index for overweight was highest among the Turkish and Moroccan children, which reveals that overweight is more severe in children of Turkish and Moroccan origin compared to children of Dutch origin. The severity of overweight increased between 1980 and 2009 in Dutch children, and between 1997 and 2009 in Turkish children, while a decrease between 1997 and 2009 was observed in Moroccan children. Part of the ethnic differences in morbid obesity rates can be explained by the educational level of the parents, as more Turkish and Moroccan children have low educated parents and morbid obesity is more prevalent among children of low educated parents.

The extended (IOTF) cut-offs encourage direct comparison of trends in child morbid obesity worldwide. The cut-offs are less arbitrary and more internationally applicable than other definitions, such as centiles on the United States (US) growth charts [17]. Until now, the prevalence of morbid obesity by the extended international (IOTF) cut-offs has only been reported in one study in New Zealand [18]. This study was limited to secondary school students and did not report information on trends. They showed that among 9,107 secondary school students in 2007, the prevalence of morbid obesity was 2.5%. Our study showed that the prevalence of morbid obesity in secondary school children of Dutch, Turkish and Moroccan origin was much lower in 2009 than the New Zealand secondary school children in 2007. Other studies applied as cut-off for extreme or severe obesity the 99^th^ percentile or a BMI≥120% of the 95^th^ percentile [19], [20] for age and sex according to the growth charts of the 2000 Center for Disease Control (CDC) [17], which complicates a fair comparison. At eighteen years, a BMI≥120% of the 95^th^ CDC growth chart corresponds to a BMI of 34.8 kg/m^2^ in boys and 36.4 kg/m^2^ in girls. A study in millions of US pre-school children living in low-income families showed that the prevalence of extreme obesity (≥120% of the P95 CDC) increased from 1.75% in 1998 to 2.22% in 2003 and decreased to 2.07% in 2010 [19]. Another study among 42,559 US children aged 3–5 years between 2007 and 2010 reported a prevalence of extreme obesity (≥120% of the P95 CDC) of 1.6% [20]. Among US children aged 2 to 19 years, the prevalence of extreme obesity (≥P99 CDC) was 3.8% [21] and in 6.9% of U.S. sixth graders in the HEALTHY cohort [22]. A study in New Zealand among 3,275 schoolchildren aged 4–15 year showed that the prevalence of extreme obesity (≥P99 CDC) was 2.7% in 2002 [23].

In agreement with our study, high differences in prevalence rates among ethnic groups within countries were reported [18]–[23]. In our study part of the ethnic differences in morbid obesity rates can be explained by the educational level of the parents. Families with low educated parents may lack resources, both economical and knowledge, which can reduce their ability to control weight gain by making healthy food choices and taking opportunities for physical activity [24]–[26]. Ethnic differences in morbid obesity may also be explained by differences in cultural opinions about body shape and acceptable weight gain [27]–[29]. Also, cultural and religious norms influencing dietary and physical activity patterns may play a role [27]–[29]. It is reported that children of Turkish origin in The Netherlands watch more TV [30], play less outdoor [31], have a higher snack intake [31], and less often have breakfast [30] compared to children of Dutch origin. All these results show that interventions or prevention programs should pay attention to the cultural aspects and educational level of the targeted population.

A particular strength of our study is the consistent methodology and inclusion/exclusion criteria, and objective measurements of height and weight in the large national growth studies between 1980 and 2009. The baseline growth study was performed in 1980, before the worldwide obesity epidemic started. Therefore, the growth studies offer a unique opportunity to monitor trends in a period of increasing levels of BMI. The data were collected from all regions in The Netherlands and are, therefore, assumed to be generalizable for the results of The Netherlands. A limitation of our study is that we only present the prevalence rates of morbid obesity in children of Turkish and Moroccan origin in 1997 and 2009, since the 1980 study did not provide a sufficiently large sample of children of Turkish and Moroccan origins. Another limitation is the limited sample size within the three age categories of the Turkish and Moroccan children that complicates a good insight into age-related trends. Further research is needed to evaluate the trends of morbid obesity within age categories of children of non-Dutch origin.

An upward trend in overweight and obesity between 1980 and 2009 was already reported in The Netherlands [3]. Our findings show upward trends in morbid obesity as well. Monitoring and reducing the prevalence of childhood morbid obesity is of high importance for these children, health care and the community. Childhood obesity leads to chronic illnesses [10], [11] and increased risk of mortality in middle age [12]. Therefore, an upward trend in morbid obesity and morbidity index for overweight may have a higher impact on health costs, than an upward trend in overweight. For this reason, it is important to monitor not only overweight, but also morbid obesity and the morbidity index for overweight. The Partnership Overweight Netherlands (PON) provides an integrated health care standard for obesity involving strategies for diagnosis and early detection of high-risk individuals as well as appropriate combined lifestyle interventions for those who are overweight and obese and, when appropriate, additional medical therapies [32].

## Conclusions

Upward trends of morbid obesity in Dutch and Turkish children in The Netherlands occurred since respectively 1980 and 1997, while this prevalence remained almost stable in Moroccan children since 1997. The Dutch and Turkish children also showed an upward trend in morbidity index for overweight since respectively 1980 and 1997, while the Moroccan children showed a downward trend since 1997. Monitoring trends of morbid obesity according to international comparable cut-offs for BMI is important, because of the great impact on health care and costs.
